# Influence of phylogenetic scale on the relationships of taxonomic and phylogenetic turnovers with environment for angiosperms in China

**DOI:** 10.1002/ece3.8544

**Published:** 2022-02-07

**Authors:** Hong Qian, Fabien Leprieur, Yi Jin, Xianli Wang, Tao Deng

**Affiliations:** ^1^ Research and Collections Center Illinois State Museum Springfield Illinois USA; ^2^ MARBEC Université de Montpellier CNRS Ifremer IRD Montpellier France; ^3^ Institut Universitaire de France (IUF) Paris France; ^4^ Key Laboratory of State Forestry Administration on Biodiversity Conservation in Karst Mountainous Areas of Southwestern China Guizhou Normal University Guiyang China; ^5^ Natural Resources Canada Canadian Forest Service Northern Forestry Centre Edmonton AB Canada; ^6^ CAS Key Laboratory for Plant Diversity and Biogeography of East Asia Kunming Institute of Botany Chinese Academy of Sciences Kunming China

**Keywords:** Chinese flora, environmental gradient, flowering plant, latitudinal gradient, phylogenetic beta diversity

## Abstract

We aim to assess the influence of phylogenetic scale on the relationships of taxonomic and phylogenetic turnovers with environment for angiosperms in China. Specifically, we quantify the effects of contemporary climate on β‐diversity at different phylogenetic scales representing different evolutionary depths of angiosperms. We sampled a latitudinal gradient and a longitudinal gradient of angiosperm assemblages across China (each ≥3400 km). Species composition in each assemblage was documented. Three metrics of β‐diversity (β_sim.tax_ measuring taxonomic β‐diversity; β_sim.phy_ and D_pw_ measuring tip‐ and basal‐weighted phylogenetic β‐diversity, respectively) were quantified among assemblages at sequential depths in the evolutionary history of angiosperms from the tips to deeper branches. This was done by slicing the angiosperm phylogenetic tree at six evolutionary depths (0, 15, 30, 45, 60, and 75 million years ago). β‐diversity at each evolutionary depth was related to geographic and climatic distances between assemblages. In general, the relationship between β‐diversity and climatic distance decreased from shallow to deep evolutionary time slice for all the three metrics. The slopes of the decreasing trends for β_sim.tax_ and β_sim.phy_ were much steeper for the latitudinal gradient than for the longitudinal gradient. The decreasing trend of the strength of the relationship was monotonic in all cases except for D_pw_ across the longitudinal gradient. Geographic distance between assemblages explained little variation in β‐diversity that was not explained by climatic distance. Our study shows that the strength of the relationship between β‐diversity and climatic distance decreases conspicuously from shallow to deep evolutionary depth for the latitudinal gradient, but this decreasing trend is less steep for the longitudinal gradient than for the latitudinal gradient, which likely reflects the influence of historical processes (e.g., the collision of the Indian plate with the Eurasian plate) on β‐diversity.

## Graphical abstract

Knowledge of the relationships between beta diversity and climate distance at different evolutionary depths (phylogenetic scales) is important to understanding species assembly through evolutionary time‐scale. A few studies have taken this approach to explore beta diversity patterns, and no such study has been reported for flowering plants (angiosperms). In our study, we analyzed angiosperm assemblages across two long gradients (one in the S‐N direction, the other in the E‐W direction, each being longer than 3400 km) in China to explore beta diversity patterns of angiosperms. Comparing patterns between the two gradients allows to determine ecological and historical (e.g., plate tectonic) effects on the patterns observed in this study.

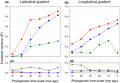

## 1 INTRODUCTION

Determining whether spatial gradients of species diversity are primarily influenced by historical and evolutionary factors or by contemporary environmental conditions continues to be a longstanding and controversial issue in ecology and biogeography (Belmaker & Jetz, 2015; Marin et al., 2018; Pellissier et al., 2014; Ricklefs, 2004). Spatial differences in species diversity can be measured through the number of species inhabiting a given locality or region (α‐diversity), but also by the change in species composition between localities (β‐diversity) (Anderson et al., 2011; Baselga, 2010). Studies on β‐diversity were found particularly useful for advancing our understanding of the regional processes shaping contemporary diversity patterns (e.g., Dobrovolski et al., 2012; Leprieur et al., 2011; Qian et al., 2020).

Beyond the taxonomic dimension of species diversity, an increasing number of studies have incorporated the phylogenetic relatedness between different species when quantifying β‐diversity (e.g., Graham & Fine, 2008; Leprieur et al., 2012; Peixoto et al., 2017; Qian et al., 2020), which has substantially advanced our understanding of the ecological and evolutionary mechanisms structuring communities (Graham & Fine, 2008; Morlon et al., 2011; Saladin et al., 2019). Analogous to taxonomic beta diversity, which measures change in species composition across space, phylogenetic β‐diversity measures the extent to which assemblages differ in terms of the evolutionary relationships of its members (Graham & Fine, 2008). However, most β‐diversity studies focus on differences in species (or lineage) composition for the present time (i.e., one time slice) or recent past using tip‐weighted metrics. Phylogenetic time‐scale is now recognized as an important issue to consider in community ecology and biogeography (Graham et al., 2018). A few studies have explored patterns of β‐diversity at different phylogenetic time‐scales from deep to shallow evolutionary histories of an interested organismal group (but see Collart et al., 2021; Cowman et al., 2017; He et al., 2018; Mazel et al., 2017). Recently, Groussin et al. (2017) and Mazel et al. (2017) proposed an approach, namely, Beta Diversity Through Time (BDTT), which computes β‐diversity between assemblages at different time periods along the phylogenetic time‐scale. This approach allows (i) separating shallow (i.e., toward the tips of the phylogeny) versus deep (i.e., toward the root of the phylogeny) β‐diversity patterns and (ii) identifying the phylogenetic time‐scale at which geographical and environmental factors have displayed the greatest influence on β‐diversity. Specifically, they found that contemporary climatic conditions were more important than geographical factors in explaining β‐diversity variation at shallower phylogenetic scales for both mammals and birds. Recently, Collart et al. (2021) came to the same conclusion for spatial phylogenetic turnover in liverworts.

Angiosperms account for ~95% of all species of vascular plants worldwide (Freiberg et al., 2020) and are important components of nearly all terrestrial habitats (Raven & Axelrod, 1974). Knowledge of how deep evolutionary histories can affect ecological assembly of angiosperms can help understand the mechanisms generating heterogeneous patterns of biodiversity across the world. China is rich in terms of angiosperms, harboring over 29,000 native species of angiosperms (Huang et al., 2013). Because China covers a great land area, spanning over 35° in latitude and 60° in longitude, it holds marked climatic gradients from the south to the north and from the east to the west (Wu, 1980). Previous studies have investigated taxonomic and phylogenetic β‐diversity of angiosperms in China (e.g., Qian et al., 2020, 2021), but no studies have investigated the influence of different phylogenetic time‐scales on the relationships of taxonomic and phylogenetic turnovers with environment for angiosperms in China. The present study fills this important knowledge gap.

This study aims at testing the hypothesis that the relationship between β‐diversity and contemporary climatic conditions is weaker at deeper evolutionary times because deeper clades are more likely to overlap in geographic or environmental space (see Mazel et al. (2017) and Saladin et al. (2019) for details about this hypothesis). Indeed, present‐day environmental conditions might not reflect deep time climate change (e.g., Eocene–Oligocene climatic optimum) and the influence of colonization history linked to plate tectonics. To test this hypothesis, this study uses regional angiosperm assemblages distributed across two climatic gradients in China: one running from the south to the north of the eastern part of China (i.e., a latitudinal gradient), the other running from the east to the west of the southern part of China (i.e., a longitudinal gradient), as shown in Appendix S1. Although temperature and precipitation decrease along both gradients (Appendix S2), these two climatic gradients, as well as species composition in regional assemblages associated with them, might have been driven by different mechanisms. The decrease of temperature and precipitation from the south to the north across the latitudinal gradient was primarily caused by global climate cooling during the Tertiary and Quaternary, forcing species at higher latitudes to migrate to lower latitudes, to evolve traits to tolerate cold and dry climate, or to become extinct (Qian et al., 2013). During glacial–interglacial cycles, species migrated back and forth along the latitudinal gradient, which would have caused mixture of species from assemblages at different latitudes across the latitudinal gradient. Thus, species in current assemblages across the latitudinal gradient would be assembled from the same species pool to a large degree.

In contrast, the decrease of temperature and precipitation from the east to the west across the longitudinal gradient was primarily caused by the uplift of the Himalayas and the Tibetan Plateau, which was in turn caused by the collision of the Indian plate with the Eurasian plate during the Eocene and has enormously affected climatic (particularly precipitation) patterns in Asia (An et al., 2001). During glacial–interglacial cycles, because species primarily migrated back and forth in the north–south direction, there was likely little mixture among species assemblages at different longitudes across the longitudinal gradient. Furthermore, because the Indian plate was part of the Gondwana supercontinent, whose flora had a substantially different evolutionary history than the flora of the Laurasia supercontinent (Raven & Axelrod, 1974), the mixture of elements of the Gondwanan paleoflora carried by the Indian plate with elements of the Laurasian paleoflora in the Himalayas would have added branches with deep evolutionary histories of Gondwanan plants in the Himalayas. Because the western end of the longitudinal gradient of this study is located in the complex of the Himalayas and the Tibetan Plateau whereas the eastern end of the longitudinal gradient is far from the complex, angiosperm assemblages in the western part of the longitudinal gradient would carry evolutionary histories of both Gondwanan and Laurasian paleofloras, whereas angiosperm assemblages in the eastern part of the longitudinal gradient might carry little evolutionary history of Gondwanan paleoflora. In other words, species in current assemblages across the longitudinal gradient might be assembled from different species pools in varying degrees. As a result, pattern and strength of weakening the relationship between β‐diversity and current environmental distance from recent to deep evolutionary times might substantially differ between the latitudinal and longitudinal gradients. In particular, one might expect that at deep evolutionary times, the relationship between β‐diversity and climatic distance would be stronger for the longitudinal gradient than for the latitudinal gradient.

## 2 METHODS

### 2.1 Species assemblages across climatic gradients

We divided China into 100 km × 100 km grid cells, as in Lu et al. (2018) and Qian et al. (2020), and assembled a latitudinal gradient and a longitudinal gradient as two environmental gradients (Appendix S1). All angiosperm species located in the two gradients were included in this study. The two gradients each were ≥3400 km long and 300 km wide (Appendix S1). The latitudinal gradient represents one of the longest thermal gradients constrained to a narrow range of longitude, while the longitudinal gradient represents one of the longest humidity gradients constrained to a narrow range of latitude in China. Although temperature and precipitation vary strongly along both gradients, temperature was correlated with latitude more strongly than was precipitation across the latitudinal gradient, whereas precipitation was correlated with longitude more strongly than temperature across the longitudinal gradient (Appendix S2). The two climate gradients formed through different mechanisms: The decrease in temperature and precipitation from the south to the north across the latitudinal gradient primarily resulted from global climate cooling during the Late Tertiary and Quaternary, whereas the decrease in temperature and precipitation from the east to the west across the longitudinal gradient primarily resulted from the uplift of the complex of the Himalayas and the Tibetan Plateau due to the collision of the Indian plate with the Eurasian plate, which generated or strengthened monsoon climate in eastern China (An et al., 2001). We divided each of the two gradients into 100 km × 300 km rectangular sites as shown in Appendix S1. The geographic distance between each pair of sites was measured as the Euclidean distance between midpoints of the sites. Angiosperm species composition for each of 100 km × 300 km rectangular sites was documented based on the data compiled by Lu et al. (2018) and Qian et al. (2020).

### 2.2 Phylogeny reconstruction

The phylogeny used in this study was generated by Qian et al. (2019), using V.PhyloMaker (function build.nodes.1 and scenario 3; Jin & Qian, 2019) as a tool and an updated and expanded version of the dated megaphylogeny GBOTB reported by Smith and Brown (2018) as a backbone. In this phylogeny, all families and 97% of the genera in our data set were resolved. Of the species present in our data set, 8834 species of were included in the megaphylogeny. For the genera and species in our data set that are absent from the megaphylogeny, V.PhyloMaker added them to their respective genera (in the case of species) and families (in the case of genera). Specifically, V.PhyloMaker set branch lengths of added taxa of a family by placing the nodes evenly between dated nodes and tips within the family. In the case of adding species to a genus, species were added to the genus as polytomies at the midpoint of the branch length of the genus. This is a commonly used approach to generate phylogenies for angiosperms (e.g., Yue & Li, 2020; Zhang et al., 2020). Qian and Jin (2021) showed that using a phylogeny generated in this way in a study of community phylogenetics is generally equivalent to using a phylogeny fully resolved at the species level in the study as long as all the families and genera in the former phylogeny are resolved. Because nearly all genera in the phylogeny used in this study were resolved and a large number of species in the phylogeny were also resolved, the results of our study are expected to be robust. Because phylogenies that are resolved at the species level for all or most species in a region are rarely available, phylogenies resolved only at the genus level have commonly used in studies on community phylogenetics (Miller et al., 2018; Molina‐Venegas et al., 2015; Segovia et al., 2020), including studies, like ours, investigating geographic patterns and climatic correlates of β‐diversity across phylogenetic time‐scale (Collart et al., 2021).

### 2.3 Metrics of taxonomic and phylogenetic β‐diversity

We used Simpson dissimilarity index (β_sim_) to measure β‐diversity between species assemblages. β_sim_ = min(*b*, *c*)/[*a* + min(*b*, *c*)], where, when taxonomic β‐diversity is concerned, *a* is the number of species shared by the two sites, *b* is the number of species unique to one site, and *c* is the number of species unique to the other site (Baselga, 2010). This index is independent of difference in species richness between the two sites under comparison (Baselga, 2010). When applied to phylogenetic β‐diversity, shared and unique species are replaced with shared and unique branch lengths, respectively (Leprieur et al., 2012). We denoted taxonomic and phylogenetic β‐diversity as β_sim.tax_ and β_sim.phy_, respectively. They vary from 0 (all species or branch lengths shared by the two sites) to 1 (no species or branch lengths shared by the two sites).

β_sim.phy_ is a tip‐weighted metric of phylogenetic β‐diversity and is more sensitive to turnover near the tips of the phylogeny (i.e., more recently diverged clades) (Swenson, 2011). In addition to using β_sim.phy_ to quantify phylogenetic β‐diversity, we also used D_pw_, which measures the mean pairwise phylogenetic distance between all species pairs in two assemblages (Swenson, 2011; Webb et al., 2008) and more heavily weights turnover of deeper nodes near and at the root of the phylogeny, compared with β_sim.phy_. D_pw_ is a basal‐weighted metric of phylogenetic β‐diversity (Qian et al., 2021; Swenson, 2011) and is measured as millions of years. Using both tip‐weighted and basal‐weighted metrics of phylogenetic β‐diversity simultaneously in an analysis (e.g., McFadden et al., 2019) would allow one to better disentangle patterns and drivers of phylogenetic β‐diversity.

### 2.4 β‐diversity through time

We used the method “β‐diversity through time” (BDTT; Groussin et al., 2017; Mazel et al., 2017) to investigate changes in the relationship of β‐diversity with geographic and environmental distances for angiosperm assemblages across the latitudinal and longitudinal gradients. This approach is to truncate the phylogenetic tree at selected evolutionary times from the tips toward the root of the phylogenetic tree (Appendix S3). At each selected evolutionary time, the phylogenetic tree is pruned (cutting off tips), with all branches younger than the selected evolutionary time being collapsed to the branches from which the younger branches descended. In other words, all branches younger than the truncating (slice) time are cutoff (Appendix S3). The geographical distribution of a tip branch of the resulting phylogeny is defined as the union (combination) of the distributions of their descending branches in the original phylogenetic tree (Borregaard et al., 2014). Details of this approach are shown in Appendix S3 (also see Groussin et al., 2017; Mazel et al., 2017). This approach is conceptually similar to the analysis of β‐diversity at different levels of a taxonomic hierarchy (e.g., species, genus, and family) (Kreft & Jetz, 2010; Lomolino et al., 2010), but it has the additional advantage of being anchored in explicit evolutionary times (Mazel et al., 2017). We sliced the phylogenetic tree at six evolutionary times (0, 15, 30, 45, 60, and 75 million years ago); we did not consider evolutionary time before 75 million years ago because few major clades of angiosperms evolved before this time slice and the vast majority of angiosperms evolved after this time slice. To explore geographic and ecological patterns for various aspects of β‐diversity at each sliced evolutionary time, we calculated the three metrics of β‐diversity between pairwise angiosperm assemblages for each gradient and related them to their respective geographic and climatic distances. Both taxonomic and phylogenetic β‐diversity metrics have been used in exploring patterns of β‐diversity across evolutionary time slices (e.g., He et al., 2020; Mazel et al., 2017); accordingly, we used both types of metrics in this study, as described above. It is important to note that this approach does not intend to estimate the geographical range of each tip branch of a phylogeny at each past time (i.e., its ancestral geographical range); it is simply based on the current geographic range of each lineage (Collart et al., 2021; Mazel et al., 2017).

### 2.5 Climate data

Previous studies have shown that the intra‐annual mean, extremes, and variability of temperature and precipitation are among the most important environmental factors that drive species distribution and diversity at large scales (Kamilar et al., 2015; Patrick & Stevens, 2016; Weigelt et al., 2015; Whittaker & Niering, 1975). Accordingly, we used the following six variables to characterize the climate of each site: mean annual temperature, annual precipitation, minimum temperature of the coldest month, precipitation during the driest month, temperature seasonality, and precipitation seasonality. We obtained values for these climate variables from the WorldClim database (http://www.worldclim.org: corresponding to variables bio1, bio12, bio6, bio14, bio4, and bio15, respectively, for mean annual temperature, annual precipitation, minimum temperature of the coldest month, precipitation of the driest month, temperature seasonality, and precipitation seasonality). The mean value of each of the six climate variables was calculated for each site using 30‐arc‐second resolution data. We used the six climate variables to calculate Euclidean climate distances between pairwise sites in this study.

### 2.6 Data analysis

We used correlation analysis and linear regression analysis to explore the relationships of β‐diversity with geographic and climatic distances. For correlation analyses, we considered a correlation (Spearman's rank correlation coefficient, *r*_s_) to be strong for |*r*_s_| > 0.66, moderate for 0.66 ≥ |*r*_s_| > 0.33, and weak for |*r*_s_| ≤ 0.33 (Qian et al., 2019). When β‐diversity was simultaneously related to geographic and climate distances, we used variance partitioning approach (Legendre & Legendre, 2012) to separate the explained variance into three parts: explained uniquely by geographic distance, explained uniquely by climate distance, and explained by geographically structured climate variation (i.e., variance jointly explained by geographic and climate distances). As previous authors (e.g., Mazel et al., 2017), we considered the variance explained by climate distance uniquely and geographically structured climate variation as the variance explained by climate distance, because climatic variables are strongly structured geographically at large scales, and thus, the effects of geographically structured climate variation should be considered as indirect effects of climate (Mazel et al., 2017).

We used the following R functions or packages to calculate β‐diversity metrics and conduct statistical analyses: betapart (Baselga & Orme, 2012), vegan (Dixon, 2003), picante (Kembel et al., 2010), and PhyloMeasures (Tsirogiannis & Sandel, 2016), and SYSTAT version 7 (Wilkinson et al., 1992).

## 3 RESULTS

**FIGURE 1:**
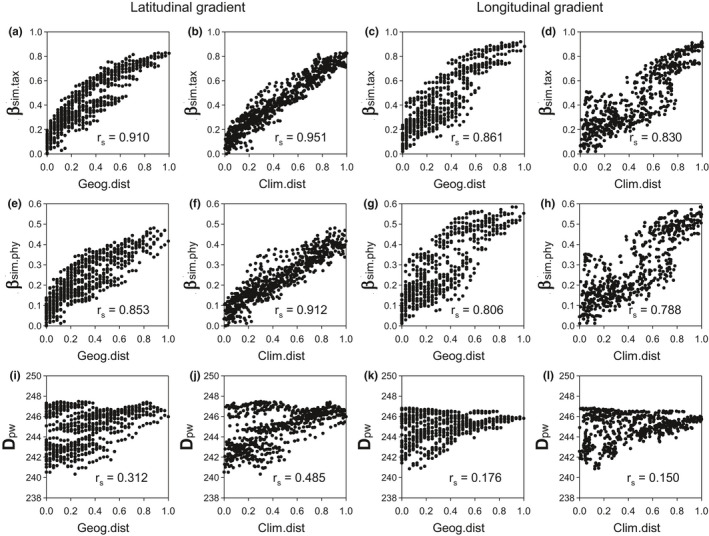
Relationships of taxonomic and phylogenetic β‐diversity (β_sim.tax_, β_sim.phy_, and D_pw_) with geographic distance (Geog.dist) and climate distance (Clim.dist) for angiosperms in sites of 100 km × 300 km along latitudinal and longitudinal transects in China (see Appendix S1 for details about the two transects). Geographic and climate distances were rescaled to vary between 0 and 1 within each transect

Taxonomic and tip‐weighted phylogenetic β‐diversity (i.e., β_sim.tax_ and β_sim.phy_, respectively) were strongly and positively correlated with both geographic and climatic distances across the latitudinal and longitudinal gradients (*r*_s_ ranging from 0.788 to 0.951; Figure 1). Correlations between β_sim.tax_ and geographic and climatic distances were stronger than those between β_sim.phy_ and geographic and climatic distances (compare panels in the first row with those in the second row in Figure 1). When the basal‐weighted phylogenetic β‐diversity (D_pw_) was concerned, its relationships with geographic and climatic distances were moderate or weak (*r*_s_ ranging from 0.150 to 0.485; Figure 1). For all the three measures of β‐diversity, their relationships with climatic distances were stronger when considering the latitudinal gradient, especially for D_pw_ (Figure 1).

**FIGURE 2:**
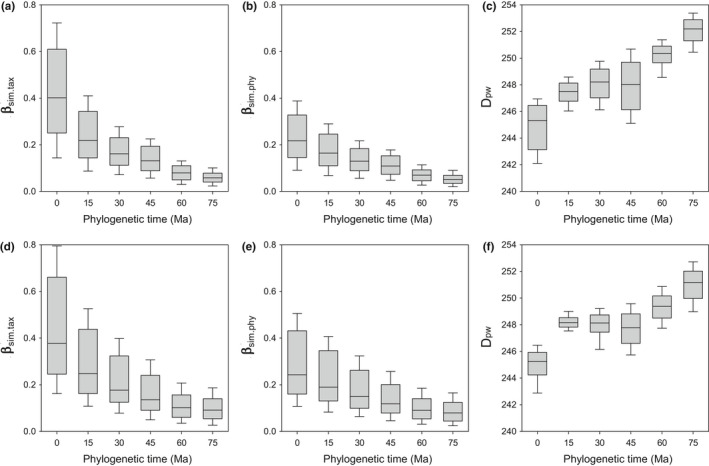
Variation of phylogenetic β‐diversity (β_sim.tax_, β_sim.phy_, and D_pw_) across phylogenetic time‐scale (millions of years ago, Ma) for the latitudinal and longitudinal gradients (the first and second rows of panels, respectively). The boxes show the median and the 25th and 75th percentiles, whereas the whiskers represent the 10th and 90th percentiles. The number of values for the analysis of each box was 630 for the latitudinal gradient and 561 for the longitudinal gradient

Both β_sim.tax_ and β_sim.phy_ decreased across the phylogenetic time‐scale from the recent to ancient times, but D_pw_ tended to increase across the phylogenetic time‐scale, regardless of whether the latitudinal or longitudinal gradient was considered (Figure 2). The trend of change in β‐diversity across the phylogenetic time‐scale was smoother for β_sim.tax_ and β_sim.phy_ than for D_pw_ (Figure 2).

**FIGURE 3:**
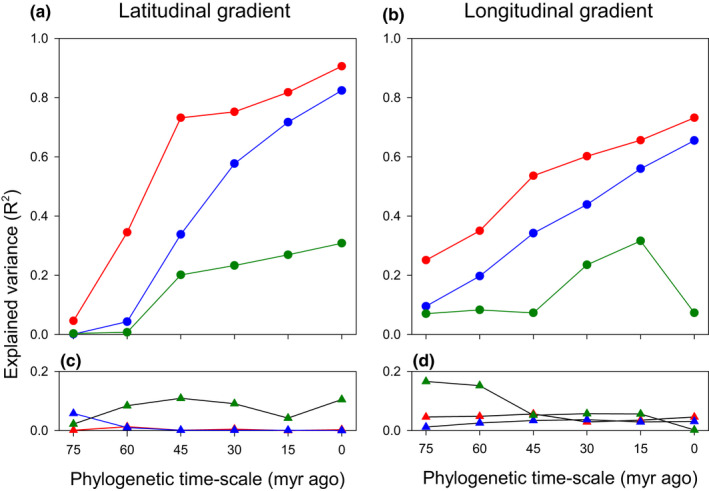
Variance in taxonomic and phylogenetic β‐diversity explained by (a and b) climatic distance (total effect of climate effect, i.e., unique climate effect plus spatially structured climate effect), and (c and d) geographic distance (unique geographic effect) at six phylogenetic depths (0, 15, 30, 45, 60, and 75 million years (myr)) for two gradients across China (a and c, the latitudinal gradient; b and d, the longitudinal gradient; see Appendix S1 for details). In each panel, red color represents taxonomic β‐diversity (β_sim.tax_), blue color represents tip‐weighted phylogenetic β‐diversity (β_sim.phy_), and green color represents basal‐weighted phylogenetic β‐diversity (D_pw_). See Appendix S4 for a detailed pattern for the relation of basal‐weighted phylogenetic β‐diversity with climatic distance across the phylogenetic time‐scale from 0 to 15 million years ago for the longitudinal gradient

The relationship between β‐diversity and climate distance became, in general, weaker at deeper evolutionary times for all the three measures of β‐diversity (β_sim.tax_, β_sim.phy_, D_pw_) for both gradients except for D_pw_ for the evolutionary time between 0 and 15 million years ago for the longitudinal gradient (Figure 3). Among the three measures of β‐diversity at each evolutionary time slice, the relationship between β‐diversity and climate distance was strongest for β_sim.tax_ and weakest for D_pw_ for both latitudinal and longitudinal gradients (Figure 2). The decrease in the strength of the relationship between either β_sim.tax_ or β_sim.phy_ and climate distance tended to be sharper for the latitudinal gradient than for the longitudinal gradient (compare Figure 2a with b).

The unique effect of geographic distance on β‐diversity was weak (*R* ^2^ < 0.10 in most cases) across the phylogenetic time‐scale regardless of whether taxonomic or phylogenetic β‐diversity was considered (Figure 3c,d; Table S1). The unique effect of geographic distance was generally stronger on D_pw_ than on β_sim.tax_ and β_sim.phy_ across the phylogenetic time‐scale (Figure 3c,d). However, the trend of the strength of geographic distance effects across the phylogenetic time‐scale tended to be in the opposite direction for the two gradients. For example, the strength of geographic distance effect decreased from 45 to 75 million years ago for the latitudinal gradient but increased from 45 to 75 million years ago for the longitudinal gradient (Figure 3c,d).

## 4 DISCUSSION

The increasing effect of climate distance on β‐diversity of angiosperms from past to recent times across the phylogenetic time‐scale of our study was found for both latitudinal and longitudinal gradients in China, which is consistent with the findings of Mazel et al. (2017) for birds and mammals at the global scale. These results are not unexpected given that (i) present‐day environmental conditions do not always reflect past conditions that shaped lineage distributions over long time periods; (ii) isolation processes associated with plate tectonics were more prevalent in the deep time (Gaboriau et al., 2019). However, we found differences in the strength of the relationship between climatic (and geographic) distance and β‐diversity between latitudinal and longitudinal gradients, which provided additional insights into our understanding of the processes shaping regional assemblages of angiosperms in China (Qian et al., 2020, 2021).

### 4.1 Influence of climatic distance

Patterns of decreasing strength in the relationship between β‐diversity and climatic distance differ between the latitudinal and longitudinal gradients. In general, the relationship was stronger for the latitudinal gradient than for the longitudinal gradient at a shallow phylogenetic depth but was weaker for the latitudinal gradient than for the longitudinal gradient at a deep phylogenetic depth, although the relationship for basal‐weighted phylogenetic β‐diversity appears to be an exception (Figure 1). Thus, the rate of decreasing strength in the relationship between β‐diversity and climatic distance is lower for the longitudinal gradient than for the latitudinal gradient.

The fact that the relationship between β‐diversity and geographic distance is much stronger at the evolutionary time period from 60–75 million years ago than at shallower evolutionary times for the longitudinal gradient (Figure 3d) suggests that the fusion of the paleoflora of the Indian plate and that of the Eurasian plate might have added some ancestral lineages of the Gondwana supercontinent to the flora of the broad region including the Himalayas and the Tibetan Plateau, which would cause an increase in β‐diversity between angiosperm assemblages located at and near the two ends of the longitudinal gradient for deep phylogenetic time slices, supporting our hypothesis (Introduction).

Our study showed that for the longitudinal gradient, the relationship between basal‐weighted phylogenetic β‐diversity (D_pw_) and climatic distance was much weaker at the 0‐million‐years‐ago (mya) time slice than at the 15‐mya time slice. This pattern is strongly inconsistent with the prediction of the hypothesis proposed by Mazel et al. (2017), although β‐diversity may be higher at a deeper evolutionary time, as shown in Figure 1 of Mazel et al. (2017) for their theoretical example of β‐diversity change through time and in Figure 2 of Collart et al. (2021) for empirical data for liverworts. For example, Collart et al. (2021) found that the relationship between liverwort β‐diversity and climatic distance is weaker at a younger geological time between approximately 30 mya and the present. To have a closer look at the variation in the relationship between basal‐weighted phylogenetic β‐diversity and climatic distance, we computed basal‐weighted phylogenetic β‐diversity for each 1‐mya time slice from 0 to 15 mya. As shown in Appendix S4, the variance in D_pw_ explained by climatic distance varied little and had no clear trend across the time‐scale from 0 to 8 mya, from which the explained variance increased monotonically and peaked at ~14 mya. It is not clear what mechanisms have caused the apparent hump‐shaped pattern for the relationship between D_pw_ and climate distance in the longitudinal gradient. One possible cause might be the geologically recent large‐scale uplift of the Tibetan Plateau and the Hengduan Mountains, particularly in the recent 15 million years (Shi et al., 1998; Xing & Ree, 2017), due to the collision of the Indian plate with the Eurasian plate during the Eocene (55–45 million years ago; Sengör & Natal'in, 1996). Because the steep climatic gradient from coastal areas in eastern China to the Tibetan Plateau in the west, which originated primarily due to the uplift of the Tibetan Plateau, is relatively young, changes in phylogenetic structure of plant communities in western China, particularly on the Tibetan Plateau, might have lagged behind climate changes due to the uplift of the plateau. This would result in a weak relationship between basal‐weighted phylogenetic β‐diversity and modern climate distance, particularly at a young phylogenetic time slice.

### 4.2 Influence of geographical distance

For both latitudinal and longitudinal gradients, the amount of the variation in β‐diversity that was uniquely explained by geographic distance is small (Figure 2). This finding from our study is consistent with that of Collart et al. (2021), who observed that for a given time slice, the correlation of liverwort β‐diversity with geographic distance is, on average, much weaker than that with climatic distance. However, our finding is contrary to that of Mazel et al. (2017), who found that the unique effect of geographic distance on β‐diversity is much stronger than the effect of climatic distance for birds and mammals across the world. This discrepancy may be because the assemblages used in our study were constrained to a single continent, whereas the assemblages used in Mazel et al. (2017) were distributed in different continents. In general, dispersal barriers would be greater between continents than within continents. Although liverwort assemblages analyzed by Collart et al. (2021) were also distributed in different continents across the world, due to the high long‐distance dispersal capacities of liverworts, dispersal limitation does not play an important role in determining β‐diversity of liverworts (Collart et al., 2021).

When taxonomic and tip‐weighted phylogenetic β‐diversity were considered, geographic distance did not explain additional variation in β‐diversity across the examined evolutionary period (i.e., 75 million years) for the latitudinal gradient, except for the time slice of 75 million years ago (Figure 2c). However, geographic distance did explain some additional variation in β‐diversity across the evolutionary period for the longitudinal gradient (Figure 2d). This suggests that geography‐related dispersal barriers have played a greater role for the longitudinal gradient than for the latitudinal gradient. As we noted above, across a longitudinal gradient, plant migration driven by glacial–interglacial cycles was in the north–south direction (i.e., along latitudinal gradients), rather than in the east–west direction (i.e., along longitudinal gradients). As a result, the composition of current angiosperm assemblages across the latitudinal gradient in the eastern part of China might have primarily driven by environmental filtering of species from the same species pool, and dispersal limitation has played little role in species assembly (Qian et al., 2020). In contrast, the composition of current angiosperm assemblages across the longitudinal gradient resulted from environmental filtering acting on different latitudinal gradients and possibly different species pools during the glacial–interglacial cycles, rather than the longitudinal gradient per se, although change in species composition is strongly related to change in climate conditions across the longitudinal gradient. More importantly, the collision of the Indian plate with the Eurasian plate has generated many high, fairly rugged mountain ranges, with deep valleys between them, running in the north–south direction in southwestern China (particularly in the region of the Hengduan Mountains), and large river systems such as Nujiang (Salween) River, Lancangjiang (Mekong) River, and Dulong River between the high mountain ranges; these high mountain ranges and deep valleys have been acting as natural barriers preventing species from eastward spreading (Li et al., 1999; Qian, 2002).

## CONFLICT OF INTEREST

The authors declare no conflict of interest.

## AUTHOR CONTRIBUTIONS

**Hong Qian:** Conceptualization (equal); formal analysis (equal); writing – original draft (equal). **Fabien Leprieur:** Writing – review & editing (equal). **Yi Jin:** Formal analysis (equal); investigation (equal); writing – review & editing (equal). **Xianli Wang:** Data curation (equal); writing – review & editing (equal). **Tao Deng:** Data curation (equal); methodology (equal); writing – review & editing (equal).

## Supporting information

Supplementary Material

## Data Availability

The data used in this study have been published. Details about data sources are cited in the article, including distributions of Chinese angiosperms in 100‐km grid cells (Lu et al., 2018; http://www.darwintree.cn/resource/spatial_data), and climatic data at WorldClim database (http://worldclim.org).
