# Supplementary material for: Influence of phylogenetic scale on the relationships of taxonomic and phylogenetic turnovers with environment for angiosperms in China
Source: Ecol Evol. 2022 Feb 7;12(2):e8544. doi: 10.1002/ece3.8544 (PMC8821769; doi:10.1002/ece3.8544)
Supplement: Supplementary file 1 — Supplementary Material [file ECE3-12-e8544-s001.doc]

**Supporting Information**

**Appendix S1.** Maps showing (a) topography, (b) the latitudinal gradient, and (c) the longitudinal gradient used in this study, which were assembled from 100 × 100 km grid cells in China.

(a)


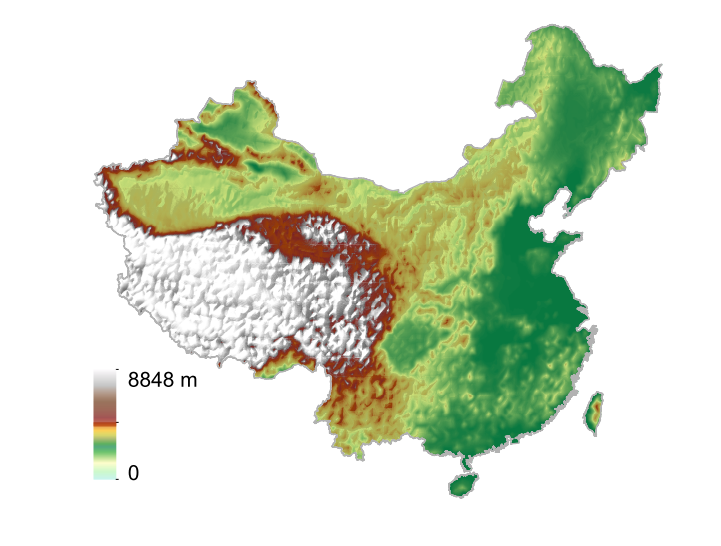


(b) (c)


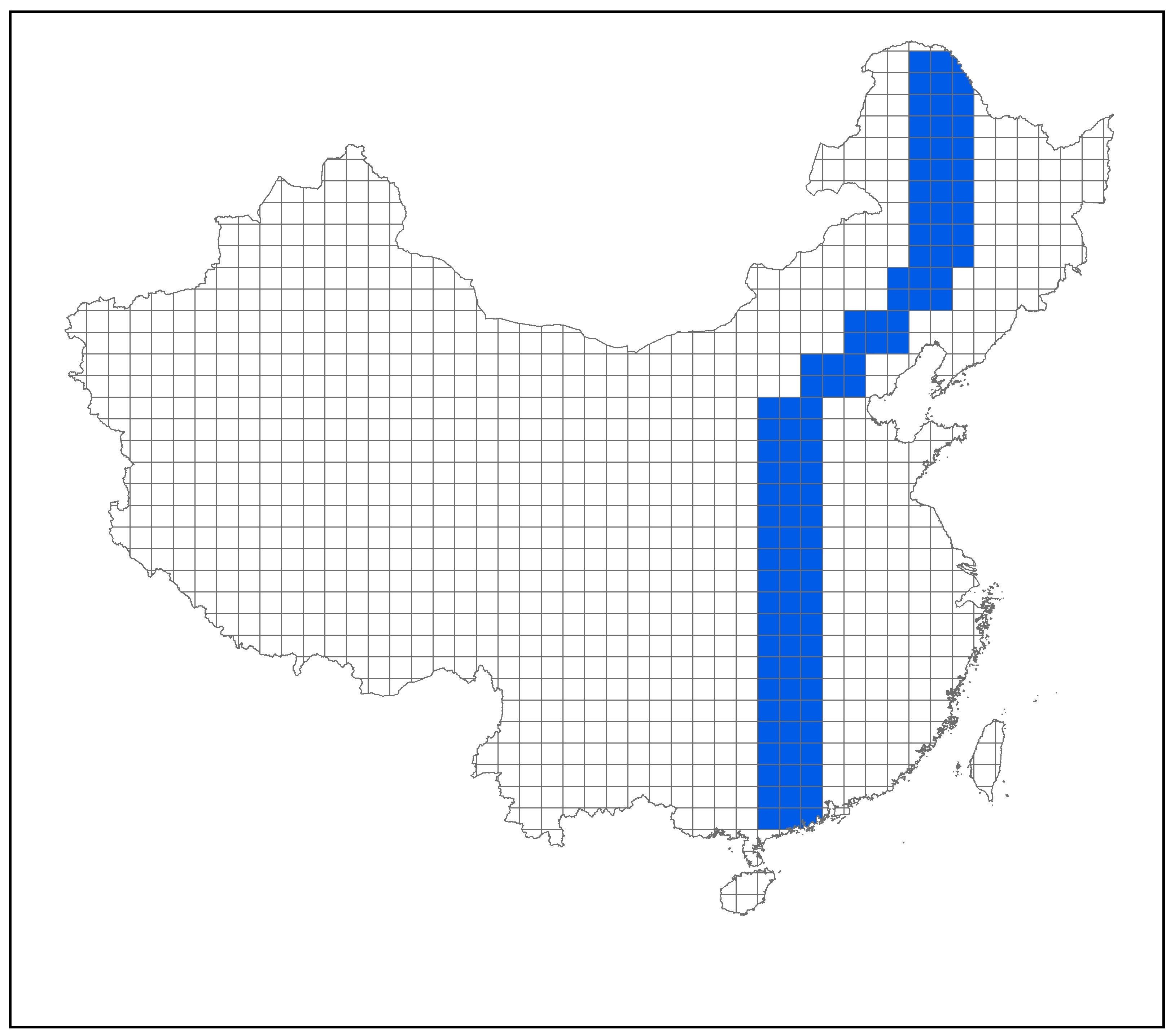

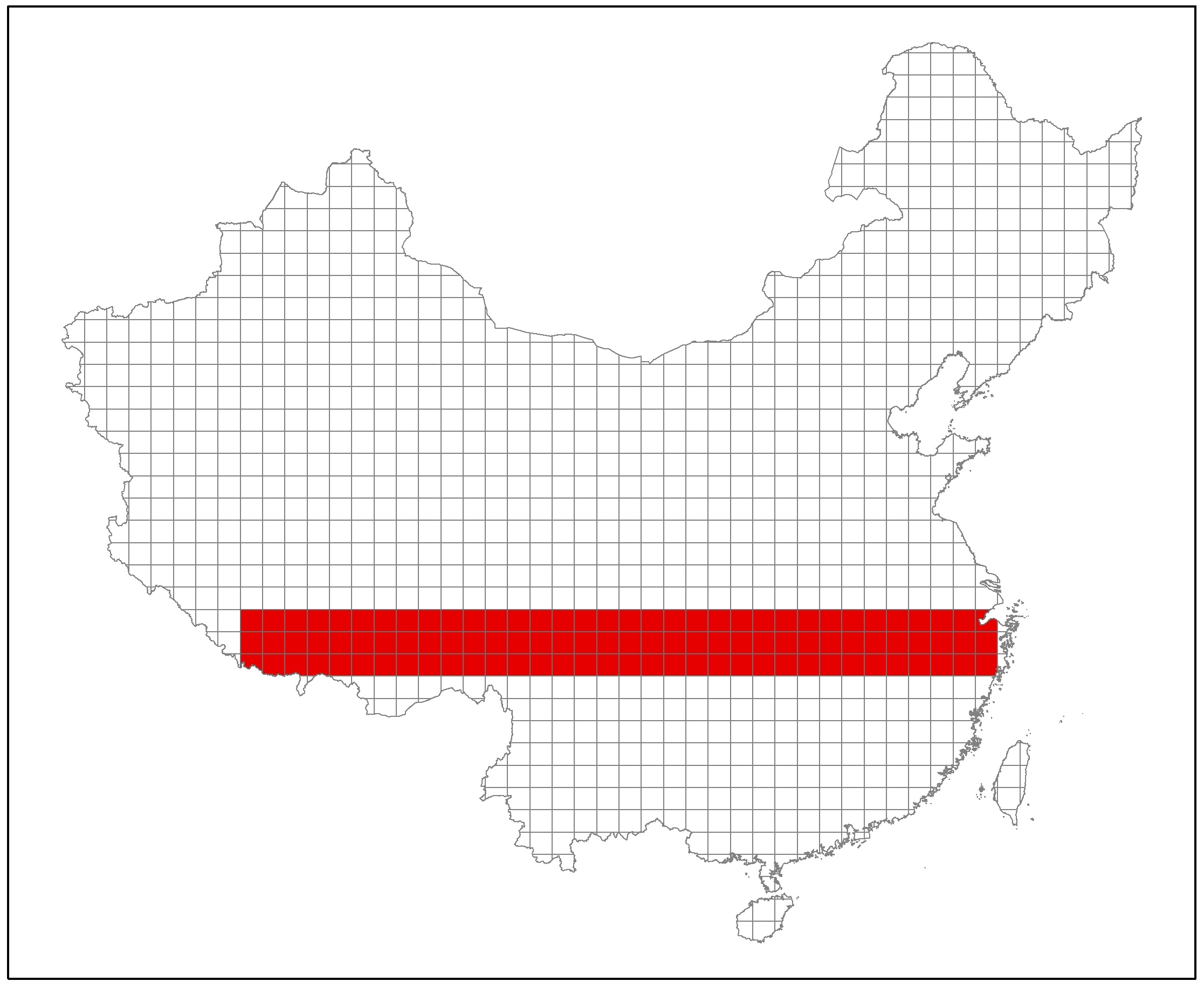


**Appendix S2.** Variation in mean annual temperature and annual precipitation along the latitudinal gradient (**a** and **b**) and the longitudinal gradient (**c** and **d**) sampled in China (as shown in Appendix S1). Red lines are simple regressions lines, indicating that the latitudinal gradient represents a strong temperature gradient whereas the longitudinal gradient represents a strong precipitation gradient.

**Appendix S3.** An example showing truncation of a phylogenetic tree at different phylogenetic time-scales.


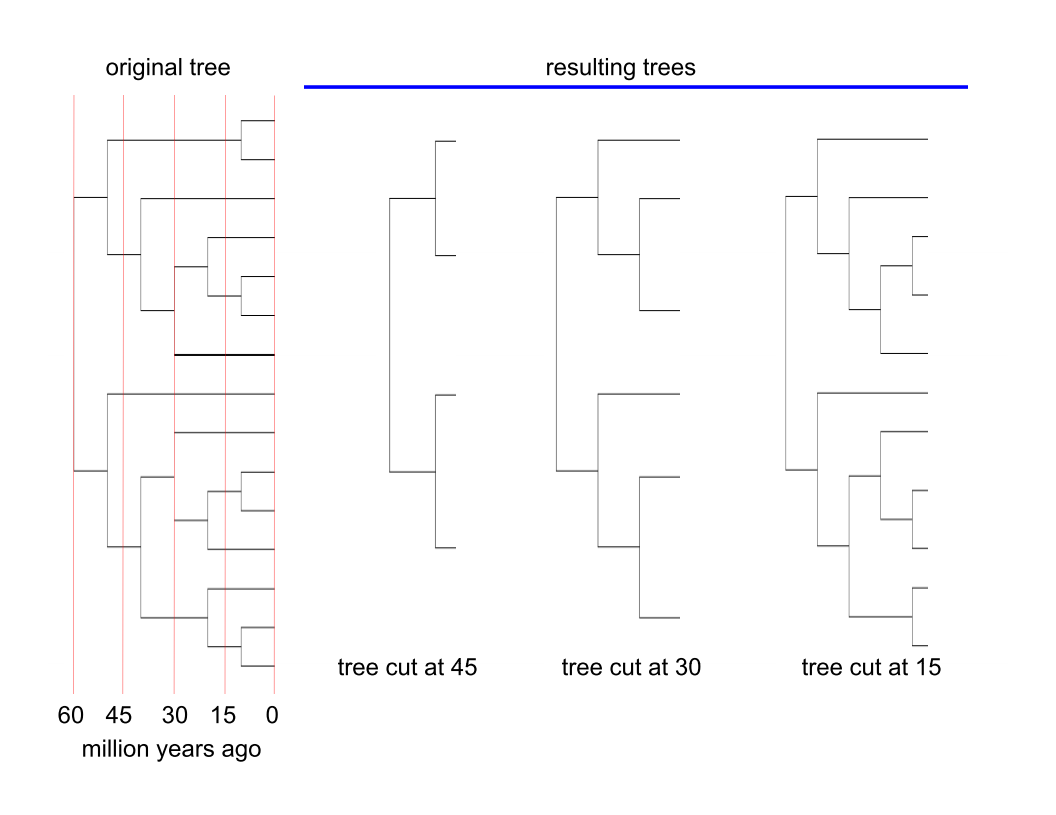


**Appendix S4**. Variance in basal-weighted phylogenetic β-diversity (D_pw_) explained by climatic distance through a phylogenetic time-scale from 0 to 15 million years (myr) ago for the longitudinal gradient (see Figure 1b for a pattern along the phylogenetic time-scale from 0 to 75 myr ago).

Table S1. Variance in taxonomic and phylogenetic β-diversity explained by geographic distance (G) only, climatic distance (C) only, and spatially structured climate at six phylogenetic depths (0, 15, 30, 45, 60 and 75 million years ago) for two gradients across China.

|  |  | Latitudinal gradient | | |  | Longitudinal gradient | | |
| --- | --- | --- | --- | --- | --- | --- | --- | --- |
| Metric | Time | G only | C only | Spatially structured C |  | G only | C only | Spatially structured C |
| β_sim.tax_ | 0 | 0.003 | 0.097 | 0.809 |  | 0.046 | 0.008 | 0.724 |
|  | 15 | 0.000 | 0.117 | 0.701 |  | 0.035 | 0.011 | 0.645 |
|  | 30 | 0.005 | 0.074 | 0.678 |  | 0.029 | 0.011 | 0.591 |
|  | 45 | 0.001 | 0.103 | 0.629 |  | 0.056 | 0.001 | 0.535 |
|  | 60 | 0.013 | 0.109 | 0.236 |  | 0.048 | 0.000 | 0.350 |
|  | 75 | 0.001 | 0.003 | 0.043 |  | 0.046 | 0.001 | 0.250 |
| β_sim.phy_ | 0 | 0.000 | 0.106 | 0.718 |  | 0.031 | 0.012 | 0.643 |
|  | 15 | 0.001 | 0.094 | 0.623 |  | 0.029 | 0.009 | 0.551 |
|  | 30 | 0.001 | 0.073 | 0.504 |  | 0.037 | 0.002 | 0.437 |
|  | 45 | 0.001 | 0.039 | 0.299 |  | 0.034 | 0.001 | 0.341 |
|  | 60 | 0.010 | 0.000 | 0.043 |  | 0.026 | 0.000 | 0.197 |
|  | 75 | 0.058 | 0.046 | -0.045 |  | 0.012 | 0.000 | 0.095 |
| D_pw_ | 0 | 0.105 | 0.263 | 0.045 |  | 0.002 | 0.003 | 0.070 |
|  | 15 | 0.042 | 0.152 | 0.117 |  | 0.056 | 0.001 | 0.315 |
|  | 30 | 0.091 | 0.216 | 0.017 |  | 0.057 | 0.003 | 0.232 |
|  | 45 | 0.109 | 0.228 | -0.027 |  | 0.052 | 0.015 | 0.058 |
|  | 60 | 0.084 | 0.090 | -0.083 |  | 0.152 | 0.072 | 0.011 |
|  | 75 | 0.022 | 0.013 | -0.010 |  | 0.166 | 0.085 | -0.015 |
